# Menstrual hygiene management in public high schools in Ghana

**DOI:** 10.4314/ahs.v22i1.11

**Published:** 2022-03

**Authors:** Patience Aseweh Abor

**Affiliations:** Department of Public Administration and Health Services Management, University of Ghana Business School, Legon-Accra

## Abstract

in the ten (10) regions of Ghana. The findings showed that most girls in Public Senior High Schools in Ghana had prior knowledge about menstruation from their mothers and sisters prior to menarche. It was revealed that majority of the girls mentioned hormones as the cause of menstruation, whilst others mentioned the uterus, bladder, vagina, and other parts of the female reproductive system. A few of them had no idea what causes menstruation. The findings again revealed that most girls use sanitary pads, while some use other materials during menstruation. Less than half of the sample of girls in this study were able to change their pads twice daily or more. About half of the girls cleansed their genital parts only when bathing during menstruation using soup and water mostly. Half of the schools used pblic tap/standpipe as their main source of water and the rest used other sources of water. Also, most schools have toilet facilities. Some useful recommendations are proffered with the aim of improving MHM in public high schools in Ghana.

## Background

The transition into adolescence is a critical moment for most girls and often comes with fear and anxiety, due to lack of knowledge and resources during menstruation and the changes that occur in the body (Sommer, 2014 & World Vision International, 2016). According to research, the age of menarche depends on geographical location, ethnic group, race and biological factors. For instance, it is argued that adolescent girls in low-income areas begin their menses between the ages of 8 and 16 (Sumpter &Torondel, 2013). Menstrual Hygiene Management (MHM) refers to ways that women cleanse, secure, use and dispose of bleeding materials (Scott et al., 2009) since it points out practical procedures for coping with monthly periods. MHM is necessary, especially in ensuring the promotion of socio-economic empowerment and growth. To tackle some challenges of MHM, the Joint Monitoring Programme under World Health Organisation and UNICEF advocated for the addition of MHM to the post 2015 sustainability goals (Sommer & Sahin 2013). In spite of this, girls in resource-poor countries still continue to use unhygienic methods to manage their menstruation as they continue to rely on cotton, wool, tissue papers and worn out cloths for their menstrual bleeding (Henry, Wall, &Kuhlmann, 2017). Also, cost of sanitary pads, lack of water supply, dirty washrooms, lack of hygienic cleansing materials all contribute to the challenges encountered by these girls (Scott et al., 2009). A report on water and sanitation from UNESCO (2014), indicate 59% and 62% primary schools in Ghana have adequate water and sanitation, respectively.

Additionally, most girls from low-middle income settings are constrained by pragmatic, social, economic and cultural factors in managing their monthly period. Sommer et al., (2014), in a study found some evidence on menstrual related experiences and challenges in a socio-cultural context. However, the study was conducted using girls in small communities from three countries (Ghana, Cambodia and Ethiopia). Jewitt &Ryley (2014) equally addressed the difficulties of managing menstruation in a school environment but focused on Kenya. In Ghana, studies such as Scott et al., (2009) have outlined MHM issues but only in some primary schools selected from five regions, whilst Dolan et al., (2013), investigated whether puberty and menstruation influence gender asymmetry in Ghana's educational system, specifically, primary and junior high school attendance.

It has been established in prior studies that, adequate management of menstrual hygiene is usually a common practice among girls in private schools, those in the cities and finally girls with knowledge on the proper utilisation of sanitary towels. However, inadequate menstrual hygiene still remains a major problem for girls and women in resource-poor countries/schools and adversely affects the health and development of adolescent girls (Henry, Wall, &Kuhlmann, 2017). It is therefore against this background that, this current study sought to investigate MHM among public Senior High School girls, unlike previous studies, which were based on primary schools and girls in their puberty ages (see Scott et al., 2009: Dolan et al., 2013). This study investigated MHM practices and its challenges among girls in public Senior High Schools across the ten (10) traditional regions of Ghana.

The rest of the paper is structured as follows: Section 2 discusses the existing literature on the subject matter, section 3 describes the methodology used in the study, section 4 includes the analysis and discussion of the results, and finally, section 5 concludes the study with appropriate recommendations.

## Literature Review

This study adopts a framework developed by The Water Supply and Sanitation Collaborative Council (WSSCC) for MHM according to Global citizen, 2015 (Water Supply & Sanitation Collaborative Council & www.globalcitizen.org, 2015). The framework includes the three interlinked dimensions for managing menstrual hygiene;

Breaking the silence: Menstruation should be seen as an aspect of the female biology and not shameful. Girls should be encouraged to discuss menstrual related issues and how it affects them emotionally and psychologically. Menstrual education and girls' openness will help prepare them physically for menstruation.

Managing menstruation hygienically and safely: Adequate water, menstrual materials, private spaces for menstrual flow management at home and in public places are essential.

Safe reuse and disposable solutions: Ensuring safe reuse and safe disposal of menstrual waste are essential for a safe environment.

## Empirical Literature

Interestingly, there have been contradictory findings in academia on the impact of access to sanitary towels on girls' absenteeism, while pointing out other key influences on attendance (Kirk &Sommer, 2006; Scott et al., 2009; Jewitt &Ryley, 2014). Henry et al (2017), assert that girls absent themselves from school during that time of the month due to lack of sanitation and water facilities in schools. In their reports on some countries including Uganda, India, Egypt, Malawi and Ethiopia, they suggest that school girls are normally absent on the first day of menstruation or during the whole period to avoid embarrassment associated with menstrual leakages. Contrarily, Montgomery et al., (2012) investigated the association between cultural constraints and high levels of poverty, inadequate water and sanitation provision, and posit that these were the issues affecting MHM in the selected countries.

Jewitt &Ryley (2014) also conducted a study in Kenya to address the difficulties of managing menstruation in school environments, whereas Dolan et al., (2013) in a similar study, investigated whether puberty and menstruation influence gender asymmetry in Ghana, specifically primary and junior high school attendance.

Knowledge and Practices of Girls in Public Schools on Menstrual Management

From 2006–2007, Sommer (2010) conducted a comparative study in rural and urban Tanzania with girls aged 16 to 19 to explore their menstrual experiences. The study found that school girls had poor menstrual and puberty knowledge. Also, in South Western Nigeria, Abioye-Kuteyi (2000) observed that the main source of menstrual knowledge was the family, hence, girls with menstrual awareness were educated by their parents, precisely mothers. On the one hand, more than 40% of the girls were ignorant of menstrual knowledge and were unable to maintain good menstrual hygiene and practices. On the other hand, 66.3 % relied on insanitary materials for their menses. This same study also found that in the Nigerian culture, it is believed that sexual issues and family life education (menstrual education and preparation) should be discussed between adults, hence, not encouraged among young girls. The results elaborated on the importance of female literacy in the society and the need for education to prepare adolescent girls psychologically before menstruation. Not Applicable.

## Abbreviations

WSSCC: Water Supply and Sanitation Collaborative Council
WASH: Water Sanitation Hygiene
MHM: Menstrual Hygiene Management
UNESCO: United Nations Educational, Scientific and Cultural Organization
UNICEF: United Nations Children's Fund
